# Symptoms of Depression and Anxiety in Adults with High-Grade Glioma: A Literature Review and Findings in a Group of Patients before Chemoradiotherapy and One Year Later

**DOI:** 10.3390/cancers14215192

**Published:** 2022-10-22

**Authors:** Monica Ribeiro, Mohamed Amine Benadjaoud, Laura Moisy, Julian Jacob, Loïc Feuvret, Alexander Balcerac, Marie-Odile Bernier, Dimitri Psimaras, Khê Hoang-Xuan, Georges Noel, Nathalie Jouniaux-Delbez, Damien Ricard

**Affiliations:** 1 Pitié-Salpêtrière-Charles Foix Hospital Group, Neuro-Oncology Departement, Sorbonne University, 75013 Paris, France; 2 Institute for Radiological Protection and Nuclear Safety, Direction of Human Health, Radiobiology and Regenerative Medicine Research Service, 92260 Fontenay-aux-Roses, France; 3 ICANS-Strasbourg-Europe Cancer Institute, Clinical Research Department, 67200 Strasbourg, France; 4 Pitié-Salpêtrière-Charles Foix Hospital Group, Radiation Oncology Department, Sorbonne University, 75013 Paris, France; 5 CNRS, ENS Paris-Saclay, Centre Borelli, Université Paris-Saclay, 91190 Gif-sur-Yvette, France; 6 CNRS, Centre Borelli, Université Paris-Cité, 75006 Paris, France; 7 Neurology Department, Percy Military Training Hospital, 92140 Clamart, France; 8 OncoNeuroTox Group, Center for Patients with Neurological Complications of Oncologic Treatments, 75013 Paris, France; 9 ICANS-Strasbourg-Europe Cancer Institute, Radiation Oncology Department, 67200 Strasbourg, France; 10 French Defense Health Service, Val-de-Grâce Medical School, 75005 Paris, France

**Keywords:** anxiety, depression, high-grade glioma, palliative care, quality of life

## Abstract

**Simple Summary:**

High-grade glioma (HGG) is the most severe type of brain cancer. At different stages of the disease, affected persons are at high risk of symptoms of depression and anxiety. If undiagnosed and untreated, these symptoms might become severe and compromise the patient’s quality of life. Improved knowledge on the prevalence, mechanisms and clinical risk factors underlying the etiology of depression and anxiety in this population is required. This may help to increase awareness on the importance of integrating consistent assessment of mood symptoms with the clinical follow-up and provide insights for developing personalized psychosocial interventions.

**Abstract:**

High-grade glioma (HGG) is associated with several external and internal stressors that may induce mood alterations at all stages of the disease. Symptoms of depression and anxiety in persons with glioma have multifactorial etiology and require active follow-up. We reviewed the literature data on the prevalence, mechanisms likely involved in the etiology of mood alterations in persons with HGG and psychosocial interventions found beneficial in treating these symptoms. We also investigated the prevalence and clinical variables that could increase the risk of depression and anxiety symptoms in a group of patients with HGG at two disease time-points: after surgery, before and 1 year after chemoradiotherapy. Literature findings revealed complex mechanisms underlying these symptoms and highlighted the importance of providing early access to palliative care. Our results show a high rate of anxiety and depression symptoms in the first stage of the disease and increased concomitance of these symptoms at the 1-year follow-up. Depression and anxiety symptoms at 1 year after the end of chemoradiotherapy were associated with the presence of symptoms at the first stage of the disease and tumor progression. Antiepileptic drugs and corticosteroid intake did not increase the risk of depressive and anxious symptoms among patients. Active management of mood alterations is an essential part of the care and contributes to patients’ well-being and quality of life.

## 1. Introduction

Gliomas are the most common type of primitive brain tumors in adults, representing 70% of all cases. Their annual incidence is 6 per 100,000 persons. High-grade gliomas (HGGs, i.e., anaplastic gliomas and glioblastomas) are classified as grade 3 and 4, respectively, according to the World Health Organization (WHO) Classification of Tumors of the Central Nervous System [1]. They account for most new cases in adults (approximately 60%) [2] and are also the most aggressive type of primary brain tumors: persons with a glioblastoma have a median survival of 14.5 months and a 5-year survival rate of 5% [3].

Receiving a diagnosis of HGG is highly stressful for the persons affected and their families. The combination of a dismal prognosis, the lack of curative treatment, the potential side effects of these treatments and the risk of disability associated with the disease can lead to significant psychological distress. Indeed, HGG may induce progressive neurological impairments, fatigue, seizures, and motor, language and cognitive deficits [4], which often compromise functional independence. Depression and anxiety are also frequent and have been identified as part of a symptom cluster associated with cancer (i.e., co-occurring and inter-correlated) that includes fatigue and cognitive impairment [5]. These symptoms have a significant impact on quality of life [6,7,8] and require active follow-up and management [9].

Each stage of the disease can be associated with potential stressors that may increase the risk of developing depression and anxiety. After the identification of the tumor on brain imaging, a biopsy or a resection is performed to determine the tumor’s histologic and molecular features and therefore provide the adequate therapy. The standard first-line treatment for HGG includes radiation therapy with concomitant and at least 6 months of adjuvant chemotherapy (temozolomide) [10]. After this treatment, patients remain under clinical and radiological follow-up until disease recurrence, when they might benefit from a second-line treatment and other lines of treatment in the disease course [11]. These stages are often accompanied by progressive subacute or acute functional deficits for which mood changes must be monitored and treated [12]. Because disease stages modulate the subjective experience, research on mood alterations in populations presenting similar disease courses can contribute to a better description of these symptoms and the development of adequate interventions [13]. However, only a few studies have investigated this topic in persons with HGG; longitudinal studies are scarce; and the prevalence of mood alterations at each stage of the disease is difficult to estimate [14].

The objective of this article is to provide an overview of the literature on the prevalence and mechanisms likely involved in both depression and anxiety in persons with HGG at different disease stages according to available data. We also provide a review of the literature on the efficacy of different strategies for managing these symptoms. Finally, we present the results of self-reported measures of depression and anxiety in a group of persons with HGG treated with radiotherapy and chemotherapy (temozolomide) and included in EpiBrainRad (NCT02544178), a cohort study currently conducted in Pitié-Salpêtrière Hospital (Paris, France) and the Institut de Cancérologie Strasbourg Europe (ICANS) (Strasbourg, France).

## 2. Review Methods

We searched entries in PubMed combining the search terms: “glioblastoma”, “malignant glioma”, “glioma”, “brain neoplasms”, “depression”, “anxiety”, “psychotherapy”, “inflammation”, “immune system”, “antiepileptic drugs”, “corticosteroid”, “radiation therapy”, “chemotherapy”, “quality of life”. We selected studies conducted in populations of persons with high-grade glioma, in heterogeneous populations and preclinical research. We also reviewed references of the selected studies that could be relevant.

For the review of prevalence rates of depression and anxiety in people with high-grade glioma, we excluded case reports, studies conducted in pediatric patients and studies including the heterogeneous population without distinction of prevalence by type and grade of tumor.

### 2.1. Depression and Anxiety in Persons with HGG

#### 2.1.1. Depression

According to meta-analyses and prospective studies, the prevalence of depression is significantly higher in persons with cancer than the general population [15,16]. This is especially the case for persons with HGG because they report greater illness intrusiveness (the extent to which disease and treatments disrupt daily activities) and lower positive affect than people with other types of cancer [17] and higher levels of psychological distress than individuals with low-grade glioma [18]. Prevalence rates reported in studies of people with glioma are highly variable, depending on assessment methods [19]. In persons with HGG, most findings are limited to the initial phase of the disease and could reach 93% in studies using self-assessment tools [6,20,21,22,23,24,25]. Longitudinal exploration of mood in these patients is challenging because of the dismal prognosis and high attrition rates. Few studies have explored depression in HGG long-term survivors (i.e., followed up for more than 2 years) and they have included small samples. Most have retrospectively explored global quality of life and have shown a rate of 30% depression symptoms in this population after 5 years [25] (Table 1).

Depressive symptoms should be screened early and monitored during follow-up to prevent the development of major depression [26]. The Hospital Anxiety and Depression Scale, the Patient Health Questionnaire and the Center for Epidemiologic Studies-Depression Scale (CES-D) [27,28,29] have been found useful in detecting individuals presenting symptoms of mood disorders and needing clinical assessment and adequate supportive care orientation [30,31].

#### 2.1.2. Depression and Glioma: Pathophysiological Processes and Effect on Prognosis

The literature suggests complex associations among several etiological processes underlying glioma growth and the development of depression. Experiencing chronic stress is a key factor because its association with inflammation has been well established. In preclinical models, chronic stressors promoted the decrease in glucocorticoid signaling and the dysregulation of the hypothalamic–pituitary–adrenal axis, which resulted in an abnormal inflammatory response that increased the risk of depression [32]. Studies show that as compared with healthy controls, large groups of people with depression show consistent and increased levels of inflammatory factors as well as associations between acute depression and a pro-inflammatory state [33]. Although not yet demonstrated in persons with glioma, this mechanism has been supported in studies showing increased prevalence of depressive syndromes in persons with autoimmune and other neurological disorders such as multiple sclerosis or stroke and elevated levels of biomarkers of inflammation [34,35].

Evidence also has shown correlations between depression and decreased overall survival in persons with glioma, including when depression occurs in preoperative phases when the tumor’s histology, grade and prognosis are unknown [21,22,36,37,38,39]. A body of research has pointed to aggravating factors underlying such associations [40,41]. Glioblastoma growth and chronic psychological distress are associated with elevated levels of inflammatory cytokines, which impair the proliferation of immune-regulator cells responsible for anti-tumor responses in mice [41,42]. Thus, neuro-inflammation might not be the only factor favoring depression in persons with cancer but might also interact with an altered immune system, which favors cancer progression [43,44].

Several pathophysiological processes impairing brain functions involved in the development of HGG include alterations of the neural immune microenvironment, angiogenesis pathways and neuron signaling. Glioma growth disrupts molecular pathways, thus leading to changes in the expression of genes and causing mutations and downregulations of proteins, notably brain-derived neurotrophic factor. This factor is involved in neuroplasticity, is thought to exert effects on glioma growth depending on the expression of its different forms [41] and is abnormally expressed in individuals with depression [40,45]. At the neural level, tumor infiltration may disrupt one of the numerous cerebral cortico-subcortical networks involved in mood regulation [46,47].

Moreover, at the behavioral level, depression is characterized by impairments in cognitive, emotional and motivational processes, with consequences on decision-goal-directed behavior and perception of reward [48]. These factors might have a negative influence on adherence to treatment regimens and the willingness to integrate psychosocial interventions that could improve mood.

#### 2.1.3. Anxiety

The etiology of anxiety disorders is closely related to the individual’s perception of threats in the environment, which may generate excessive and persisting worry over some events and activities. Cancer disease and treatments are associated with several risks, representing potential threats that may trigger anxiety symptoms in individuals at all stages of the disease, with consequences on everyday functioning and treatment adherence. Although highly associated with depression, anxiety is strongly and independently associated with mental health and quality of life in persons with cancer [49]. Symptoms of anxiety have been associated with worse emotional well-being in individuals with newly diagnosed HGG but also in their relatives [8]. To our knowledge, there are no data on the prevalence of clinically diagnosed anxiety specifically in persons with HGG; however, high levels of anxiety symptoms have been reported in this population early in the disease course as well as in long-term survivors [13,50]. Rates of anxiety symptoms according to self-reported measures could reach 55% less than 12 months after the diagnosis [23,51]. Individuals would be especially vulnerable to anxiety during the first stages of the disease because they may have difficulties coping with the diagnosis, prognosis, disability and side effects associated with cancer treatments [13,52,53].

Anxiety may affect not only quality of life but also clinical outcomes: low levels of anxiety could drive individuals’ motivation to adhere to treatments, but excessive anxiety can lead to avoidance behaviors toward routine exams and treatments [54]. The early exploration of this symptom using brief screening tools such as the Goldberg Scale [55] and the Hospital Anxiety and Depression Scale [30] can help clinicians identify abnormal levels of anxiety and treat these symptoms in order to prevent such consequences.

#### 2.1.4. Risk Factors of Depression and Anxiety

Tumor grade, a history of psychiatric disorders, neurological deficits, functional and cognitive impairment, comorbidities and lower level of education all significantly increase the risk of developing depression and anxiety symptoms [9,51,56,57]. Anxiety disorder after the diagnosis of cancer is often the reactivation of a pre-existing condition; therefore, a history of anxiety is a major risk factor. Other risk factors include the symptoms of the disease and their functional impact [54]. Cardiovascular comorbidities such as hypertension, dyslipidemia and diabetes should also be considered because they have been frequently associated with mood disorders in clinical populations [58,59].

#### 2.1.5. Oncological Treatments and Mood Alterations

Understanding the effects of oncological treatments on the mood of persons with glioma is challenging because pathophysiological mechanisms and psychological distress are both preexisting risk factors [26,41]. However, improved knowledge and awareness of treatment-related toxicities that could increase such risk are needed. Besides standard treatment protocols, other treatments may be necessary for managing associated conditions such as epilepsy because a large proportion of persons with HGG (30% to 50%) experience seizures [60]. Valproic acid and levetiracetam are frequently used and studied across populations with epilepsy, but levetiracetam use in persons with a brain tumor has been increasing over the last years given its efficacy, limited interactions with chemotherapeutic agents and limited adverse effects on cognitive functions [60,61]. Valproic acid use is associated with reduced risk of psychiatric symptoms, but levetiracetam use in epilepsy has shown increased psychiatric and behavioral side effects [62] and increased risk of drug-treated anxiety in mixed cohorts of persons with glioma [63]. However, these results have not been consistently replicated [64,65].

Glucocorticoids are often used because they provide significant clinical improvement of neurological symptoms resulting from peritumoral edema [66]. The neuropsychiatric effects of corticosteroids, notably their associations with depression, have been well demonstrated across clinical populations [67]. However, mood disturbances could be dose-dependent and reversible after treatment interruption [66,68,69].

To our knowledge, there are no clear associations between radiotherapy and depression or anxiety symptoms in persons with glioma [70]. However, brain irradiation has been shown to cause neuroinflammation and brain dysfunction [71,72,73]. Among the suspected pathophysiological mechanisms underlying radiation-induced neurotoxicity, the inhibition of neurogenesis is a major hypothesis [73] that has been investigated in studies using preclinical models. Antidepressants increased adult hippocampal neurogenesis, and increased neurogenesis reduced depression and anxiety-like behaviors in mice [74]. Accordingly, radiation combined with chemotherapy could significantly decrease hippocampal neurogenesis and dysregulations in the serotonin axis within the hippocampus [75,76]. These alterations were associated with depression and anxiety-like behaviors in mice without brain tumors, persisting 15 weeks after whole-brain irradiation [75].

The combination of chemotherapy and radiotherapy potentially increases toxicity and long-term brain dysfunction [73] and therefore increases the risk of mood alterations. Indeed, in preclinical models, the use of temozolomide alone was significantly correlated with inhibiting adult hippocampal neurogenesis as well as increasing corticosterone responses that are triggered in situations of acute stress [77].

#### 2.1.6. Therapeutic Interventions for Depression and Anxiety

Studies assessing the efficacy of psychosocial interventions on depression in persons with different types of cancers have shown positive results [78]. In these populations, a meta-analysis of randomized controlled trials assessing the efficacy of pharmacological and non-pharmacological interventions (i.e., cognitive behavioral therapy, interpersonal therapy and other approaches) showed that both approaches could be used and would have benefits [79]. Despite significant morbidity and impact on quality of life, only a few randomized controlled trials have assessed the effects of psychotherapy and more broadly, psychosocial support interventions specifically in persons with brain tumors [30]. A controlled trial showed significant effects of a multimodal psychosocial intervention, including modules of psychoeducation, neuropsychological feedback, cognitive rehabilitation, psychotherapy and couple and family support, tailored to participants’ needs. Six months after its completion, levels of depression, anxiety and stress were significantly lower and quality of life was better among participants as compared to pre-intervention levels [80]. No randomized controlled trial has addressed the efficacy of pharmacological interventions in persons with brain tumors [81].

For anxiety, therapeutic education provides information on treatments during the first phases of the disease, helping individuals to reduce anxiety associated with the side effects of their treatments and improving self-efficacy in managing them. Randomized controlled trials have shown the benefits of techniques based on cognitive behavioral interventions for stress management and for the development of effective coping strategies in persons with cancer that should also benefit those with HGG. There are several psychotherapy approaches that can be tailored to the person’s needs, which is highly recommended in case of severe anxious disorders [54].

## 3. Longitudinal Changes in Mood in Persons with HGG: Results of the EpiBrainRad Study

### 3.1. Materials and Methods

Here, we present results from the EpiBrainRad study [82] being performed in Pitié Salpêtrière Hospital and ICANS. The main objective of this prospective longitudinal study is to explore cognitive status after chemoradiotherapy and the mechanisms underlying cognitive changes in persons with HGG. Assessments include studies of dosimetric data, brain MRI features, analysis of specific biomarkers, a comprehensive cognitive evaluation and self-reported measures of depression, anxiety, fatigue and quality of life as previously described. Assessments take place after surgery and before chemoradiotherapy and once a year during 3 years of follow-up. Inclusion criteria are (1) histologically confirmed diagnosis of WHO grade 3 or 4 primary brain tumor after tumor resection or biopsy and being scheduled for a chemoradiotherapy regimen according to the Stupp protocol [10] as suggested by the neuro-oncology multidisciplinary team meeting; (2) age ≥ 18 years; (3) treatment and clinical follow-up performed in the participating hospital; and (4) speaking French fluently. The exclusion criteria are any condition likely to impair cognition (i.e., stroke, severe brain injury and history of psychiatric disorders) and a history of whole-brain radiotherapy. The study protocol was conducted according to the Helsinki principles and approved by Ethics Committee Protection des Personnes Paris VI, Ile de France (ID: CPP132/14). All participants gave their signed informed consent.

Patients reported symptoms of depression on the CES-D [83] and anxiety on the Goldberg Scale [55] after surgery and before chemoradiotherapy (T1) and 1 year after chemoradiotherapy completion (T2). The threshold used for depression symptoms was ≥16 on the CES-D and ≥5 for anxiety symptoms on the Goldberg Scale.

### 3.2. Statistical Analysis

We performed Fisher’s exact test and t-tests to assess differences in sociodemographic factors between T1 and T2 scores on self-reported measures and multivariate analysis to explore clinical variables associated with depression and anxiety symptoms. Selection of clinical variables as a candidate for the multivariate analysis was based on the Wald test from univariate logistic regression (*p*-value cut-off point of 0.15). Variable selection for the multivariate regression was based on Akaike information criterion [84]. For all analyses, the significance level for associations was *p*-value < 0.05. These variables were antiepileptic drugs (AEDs) and corticosteroids intake, disease recurrence and cardiovascular risk factors, namely hypertension, dyslipidemia and diabetes. Odds ratios (ORs) and 95% confidence intervals (CIs) were estimated. Statistical analyses involved using R (version 4.0) software [85].

### 3.3. Results

We included 229 patients between April 2015 and September 2019: 220 had a diagnosis of glioblastoma (96.0%) and 9 WHO grade 3 glioma (3.9%) whose molecular profile required chemoradiotherapy, as determined by the interdisciplinary team meeting [10]. The median Karnofsky Performance Score was 90% (Table 2). Overall, 160 (69.9%) patients underwent tumor resection, which was complete in 114 (49.8%) and partial in 46 (20.0%); 69 (30.1%) had a biopsy.

#### 3.3.1. Baseline Assessment (T1)

At T1, of the 229 patients enrolled in the study, 154 returned at least one of the questionnaires, 153/229 (66.8%) completed the CES-D and 140/229 (61.1%) completed the Goldberg scale (Table 2). Among them, 92 (60.1%) were receiving an antiepileptic drug (AED); 89 (96.7%) had levetiracetam and 48 (31.2%) were receiving corticosteroids. Additionally, 32 (20.9%) patients had hypertension, 30 (19.6%) had dyslipidemia and 9 (5.9%) had diabetes (Table 2).

**Figure 1:**
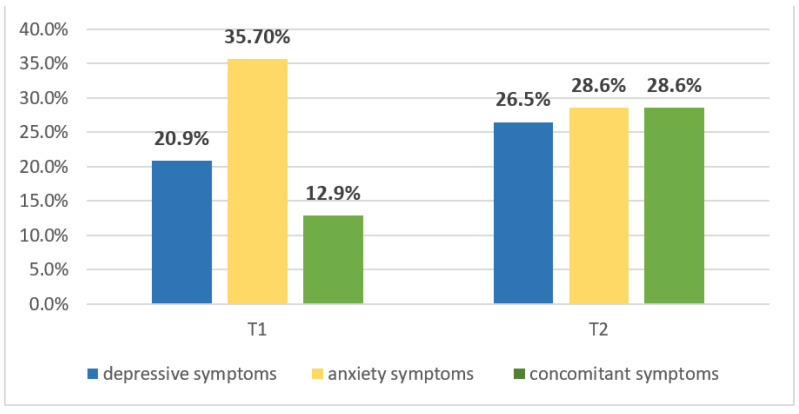
T1, after surgery and before chemoradiotherapy; T2, 1 year after chemoradiotherapy completion.

In total, 83/154 (53.6%) patients had at least one depression or anxiety symptom; 32 (20.9%) had symptoms of depression and 50 (35.7%) symptoms of anxiety (Figure 1). Among the 140 participants completing both questionnaires, 18 (12.9%) had concomitant depression and anxiety symptoms. Figure 1 shows prevalence of symptoms of depression and anxiety among participants in the EpiBrainRad study at T1 (*n* = 154) and T2 (*n* = 49).

#### 3.3.2. Follow-Up Assessment (T2)

A follow-up assessment (T2) was performed at a mean of 16.51 ± 1.72 months after baseline assessment and included 78 patients. We could not assess 130 patients because of clinical aggravation (49 cases, 37.7%), tiredness (6 cases, 4.6%) or death (75 cases, 49.7%). The remaining 21 patients had been recently enrolled (less than one year after T1). The median KPS was 90%. The mean age was significantly lower as compared with baseline; otherwise, sociodemographic and clinical characteristics did not significantly differ between T1 and T2 (Table 2).

At T2, 49/78 (62.8%) patients had CES-D and Goldberg scores. Overall, 14 (28.6%) had scores indicating at least a depression or anxiety symptom; 13 (26.5%) had symptoms of depression and 14 (28.6%) had symptoms of anxiety, which represented 92.9% of concomitant symptoms among participants presenting at least one symptom. In the whole group of patients with CES-D and Goldberg scores at T2, the concomitance of depression and anxiety was 26.5% (Figure 1).

Among the 49 participants, 31 (63.3%) were receiving an AED, 28 (90.3%) of whom were receiving levetiracetam, and 17 (34.7%) were receiving a corticosteroid. Eight (16.3%) patients had hypertension, five (10.2%) had dyslipidemia and two (4.1%) had diabetes. In total, 12 (24.5%) had disease recurrence at some point during the follow-up and 37 (75.5%) had stable disease according to the Response Assessment in Neuro-Oncology group criteria [86].

Prevalence of depression and anxiety did not differ at T1 and T2 according to scores on the CES-D (*p* = 0.433) and the Goldberg Scale (*p* = 0.387).

Some clinical variables were significant predictors of anxiety and depression symptoms at T2: mood symptoms at T1 (CES-D: OR 54.23, 95% CI 1.83–1604, *p* = 0.0184; Goldberg: OR 13.79, 95% CI 1.02–186.5, *p* = 0.043) and tumor progression (CES-D: OR: 21.28, 95% CI 1.76–256.2, *p* = 0.014; Goldberg: OR 14.23, 95% CI 1.48–136.0, *p* = 0.019). Although the association was not significant, the probability of depression and anxiety symptoms was lower among participants receiving than not receiving AEDs (CES-D: OR 0.06, 95% CI 0.003–1.05, *p* = 0.056) and anxiety symptoms (Goldberg: OR 0.08, 95% CI 0.006–1.10, *p* = 0.059).

Univariate regression analysis did not show significant associations between corticosteroids intake and mood at T1 (CES-D: *p* = 0.148; Goldberg: *p* = 0.874) or T2 (CES-D: *p* = 0.0.807; Goldberg: *p* = 0.874). It did not yield significant results for cardiovascular risk factors for CES-D (hypertension: *p* = 0.435; dyslipidemia: *p* = 0.584; diabetes: *p* = 1.000) or Goldberg score (hypertension: *p* = 0.347; dyslipidemia: *p* = 0.196; diabetes: *p* = 1.000).

## 4. Discussion

Findings in the literature have shown that depression and anxiety greatly affect quality of life and may compromise prognosis; they require active management in persons with HGG. Across studies, the cause of these symptoms is multifactorial: these individuals are prone to experience the symptoms at all stages of the disease. Results from our study of a prospective cohort of adults with HGG show that (1) after surgery and before chemoradiotherapy (T1), 53.6% of participants had at least depression or anxiety symptoms—20.9% symptoms of depression, 35.7% symptoms of anxiety and 12.9% concomitant symptoms; (2) 1 year after the completion of chemoradiotherapy (T2), the prevalence of depression and anxiety symptoms was 28.6% and 26.5%, respectively, with 28.6% of individuals presenting concomitant symptoms; (3) the probability of presenting symptoms at T2 was associated with presenting symptoms at baseline; (4) although the association was not significant, the probability of depression symptoms was lower among participants receiving than not receiving AEDs; (5) tumor progression significantly predicted anxiety and depression symptoms 1 year after chemoradiotherapy.

The literature on the prevalence of symptoms and clinically diagnosed mood disorders is inconsistent. One of the reasons is the heterogeneity of populations included in studies, both in terms of grades of brain tumor and disease stages. Yet, these are central aspects influencing the subjective experience of persons with glioma during the course of the disease. In our study of persons with HGG, we found high rates of mood symptoms among participants at the first stage, in line with some previous studies [6,20,21]. Rates of anxiety symptoms reported by our participants were higher than those of depression, which has been observed particularly at the first stages of the disease course [6,52]. This observation might be related to knowledge of the diagnosis, risks, and secondary effects of first-line treatment, which are potential triggers of anxiety states [57].

The evolution of mood alterations during the course of the disease is an important question, and longitudinal studies investigating these symptoms in homogeneous populations are scarce. In our study, the high prevalence of mood symptoms among our participants assessed at baseline remained until 1 year after initial follow-up. Importantly, our results show high concomitance of these symptoms at T2, thus suggesting worse mood as compared to T1. In line with other studies within mixed cohorts [87], our results show that depression and anxiety symptoms at baseline were independent predictors of symptoms 1 year after first-line treatment completion, which highlights the importance of the early assessment of mood symptoms. Concerning clinical variables, cardiovascular risk factors did not predict mood status at any assessment, probably because of the small number of individuals presenting these comorbidities, notably at T2. However, tumor progression strongly predicted anxiety and depression symptoms among participants at the follow-up assessment, independent of mood status at baseline. This result agrees with findings of Piil et al., showing a similar association in 87.5% of their patients with HGG presenting depression 1 year after the diagnosis [6]. Tumor progression represents a critical phase in the illness trajectory because it may reactivate high levels of psychological distress after a few months of disease stability. Mood changes might result not only from the psychological and functional impact but also from tumor-related alterations of biological factors that impair brain functioning. This is an important aspect to consider for the design of studies exploring clinical variables and mood alterations in persons with a brain tumor because results obtained in mixed populations might not reflect specific needs in terms of palliative support [13,88].

Concerning the effect of treatments on mood, our results are contradictory. Although the association was not significant, the probability of depression and anxiety symptoms was lower among participants receiving than not receiving AEDs whereas corticosteroid intake had no effect on mood. Most of our participants were taking levetiracetam, which has been associated with depression symptoms in studies among persons with epilepsy [62] and with increased use of drugs to treat anxiety in persons with different tumor types [63]. However, these results have not been consistently replicated across populations [64,65]. Some evidence suggests increased risk of depression in individuals receiving corticosteroids [66,68], whereas other studies have not shown an effect on symptoms of distress [31]. Beyond the fact that the number of participants taking these treatments was relatively small, to our knowledge, our study is the first to assess this association in a homogenous cohort of persons with HGG, which may also explain the discrepancies with literature findings. Moreover, considering that the origin of mood symptoms is multifactorial and closely related to disease symptoms and disability, the control of epilepsy and neurological symptoms could modulate this association in persons with high levels of symptom burden (i.e., persons with control of these symptoms may assess their mood status more positively as compared to the first stage of the disease). Other studies of homogeneous and larger groups are necessary to confirm these associations.

Our study presents limitations. The prevalence of depression symptoms at the follow-up might have been underestimated. At T1 and T2 assessments, the majority of participants had KPS scores above 60%, indicating that they were able to perform normal activity because the Stupp protocol is not recommended for patients with KPS < 60. Indeed, at T2 we could not assess a large proportion of participants because of clinical aggravation and fatigue, while disease symptoms might increase the risk of suffering from mood alterations [31,89]. This raises questions about the frequency of assessments and the follow-up of participants in longitudinal studies. In our study, depression and anxiety symptoms were screened at a one-year interval, along with neuropsychological assessments. This might be too long for studies exploring depression and anxiety as primary outcomes. Disease recurrence and changes in treatment lines are crucial stages but the assessment of these symptoms remains challenging at these time points, explaining the high attrition rates reported in other studies (see Table 1). Nonetheless, our results show that depression and anxiety symptoms may be frequent even among individuals with better functional status, showing that mood alterations might occur in the absence of disability.

Collectively, and in the light of the literature findings, our results show significant levels of depression and anxiety symptoms in participants from early phases of the disease that might be long-lasting. In this context, promoting early access to supportive care interventions (e.g., provided by palliative care teams) could help patients to cope with the disease and contribute to their quality of life. Palliative care provides complementary support in symptom management, information, physical and psychosocial support. Its early application has been found beneficial for quality of life, with positive effects on the duration of survival in other types of cancer [30,90]. The results may be highly valuable to help persons with HGG cope with chronic stressors and foster the prevention of mood disorders.

Little research has focused on mood disorders in long-term survivors with HGG. Describing changes in cognition and other central endpoints such as depression and anxiety symptoms, fatigue and quality of life using specific measures over 3 years in this population is among the objectives of the EpiBrainRad study. Exploring the associations between mood alterations among participants and irradiation doses to brain regions involved in cognition and mood is one of the main objectives. Results might contribute to a better understanding of the potential etiological mechanisms involved in mood but also in functional decline.

## 5. Conclusions

Depression and anxiety symptoms are frequent during the illness trajectory of persons with HGG and require consistent monitoring. The literature shows high rates of depression and anxiety symptoms among these individuals; however, the prevalence is likely underestimated because persons presenting disability (i.e., aphasia, severe cognitive deficits) are not often included in studies and their assessment for research purposes remains challenging. To better identify the needs of patients in terms of supportive care, studies investigating mood disorders in persons with glioma should include homogeneous populations because clinical characteristics and the disease phase seem to be critical modulators of mood. Different options of tailored psychological interventions can benefit persons with HGG and their relatives and significantly improve quality of life.

## Figures and Tables

**Table 1:** Prevalence of depression and anxiety symptoms in persons with high-grade glioma, according to self-reported measures from the literature.

References	*n*	Type of Study	Follow-Up Time Point	% Depression	% Anxiety
**Assessment at diagnosis**					
Litofsky et al. [22]	598	Longitudinal	Early post-operative period	93%	NA
Dehcordi et al. [20]	56	Cross-sectional	Operative period	35%	NA
Piil et al. [6]	30	Longitudinal	Early postoperative period	14%	39%
Noll et al. [21]	102	Cross-sectional	Early postoperative period	27%	NA
Ståhl et al. [24]	63	Longitudinal	Early postoperative period	17%	22%
**Assessment during the first year**					
Litofsky et al. [22]	598	Longitudinal	6 months	90%	NA
Lucchiari et al. [23]	73	Cross-sectional	<12 months	39%	55%
Piil et al. [6]	18	Longitudinal	One year	6%	6%
Ståhl et al. [24]	12	Longitudinal	One year	8%	8%
**Assessment over 2 years**					
Steinbach et al. [25]	10	Cross-sectional	>5 ans	20%	10%

**Table 2:** Sociodemographic and clinical data of participants in the EpiBrainRad study.

	T1 (*n* = 154)	T2 (*n* = 49)	*p* Value
Age, years, mean (SD) (range)	57.4 (9.3) (20–80)	53.9 (9.2) (23–74)	0.037
Sex F/M, *n* (%)	48 (31.2%)/106 (68.8%)	15 (30.6%)/34 (69.4%)	1.000
Education, years: ≥12	109 (70.8%)	39 (79.6%)	0.271
KPS ^1^, median (range)	90% (50–100)	90% (60–100)	0.548
Corticosteroids use	47 (31.4%)	17 (34.7%)	0.599 0.738
Antiepileptic drugs use	92 (60.1%)	31 (63.3%)	
Hypertension	32 (20.9%)	8 (16.3%)	0.544 0.192 1.000
Dyslipidemia	30 (19.6%)	5 (10.2%)	
Diabetes	9 (5.9%)	2 (4.1%)	

^1^ KPS, Karnofsky Performance Status.

## Data Availability

The data can be shared upon request.
